# Soil Quality Enhances Seed Germination Success in *Ephedra major*—A Pilot Experiment

**DOI:** 10.3390/plants12030438

**Published:** 2023-01-18

**Authors:** Kevin Cianfaglione, Florin Crișan, Dan Gafta

**Affiliations:** 1 Faculté de Gestion, Économie et Sciences (FGES), Université Catholique de Lille, F-59000 Lille, France; 2 Department of Taxonomy and Ecology, 3B Centre, Babeș-Bolyai University, 42 Republic Street, 400015 Cluj-Napoca, Romania

**Keywords:** central Apennines, woodland humus, garden experiment, germination rate, herbivores, single mother plant, seedlings, soil type, watering

## Abstract

There are currently knowledge gaps in the environmental context related to successful seed germination of *Ephedra major*. Therefore, we herein explore the influence of soil quality and water availability on the germination performance through a garden experiment that mimics natural site conditions. One hundred seeds were extracted from fifty ripe strobili collected randomly from the ramets of a single female plant. Ten seeds per pot were sown in ten pots, which were equally split by receiving different watering treatments (watered versus control) and soil types (S0–shallow and stony; S1–like S0 but slightly deeper; S2–like S0 but even deeper and rich in woodland humus; S3–clay-layered alluvial; S4–anthropogenic). No significant interaction effect was detected between the two manipulated factors. Watering only had a marginal effect on the germination rate, but the latter was significantly higher in S2 when compared to the other soil types. These outcomes suggest that soil quality is more important than moisture for the germination success. Its rate is expected to be higher under the open canopy of woodlands compared to open rupicolous habitats, since seeds can benefit from higher humus availability and reduced evapotranspiration.

## 1. Introduction

*Ephedra major* Host. is the second-most widespread species in Europe, covering almost the whole Mediterranean basin, but also occurring in the Middle East and central Asia [1]. In Italy, this species is sparsely found along the eastern escarpment of the central and southern Italian Peninsula as well as in the two major islands, Sicily, and Sardinia [2]). *E. major* was mentioned as a rare, relictual taxon in relation to its very fragmented distribution in Italy [3,4]. The older name *Ephedra nebrodensis* is generally considered a synonym of *Ephedra major* [5,6,7], but there are recent proposals for maintaining the former name [8,9]. Moreover, the taxonomic and phylogenetic position of the *Ephedra* species is still under debate [10].

*Ephedra major* is a dioecious species whose female individuals produce fleshy strobili, almost completely covering the seed(s). These berry-like cones are highly appreciated by insects that can disperse the seeds [11].

At a European level, *Ephedra major* subsp. *major* is currently considered a diagnostic taxon of the classes *Festuco hystricis-Ononidetea striatae* Rivas-Martinez et al. 2002 (including dwarf scrub on calcareous substrates of the Iberian Peninsula, the Western Alps and the Apennines) and *Quercetea ilicis* Br.-Bl. ex A. Bolòs et O. de Bolòs in A. Bolòs et Vayreda 1950 (pine and oak forests and associated macchia of the Mediterranean) [12]. The dominance of *E. major* in certain scrub communities could be determined by its allelopathic effect on other plant species [13].

Apart from the high capacity for vegetative multiplication due to root suckers, very little is known about the environmental context related to successful sexual reproduction in *E. major* and nothing about seed germination under natural conditions. As in other *Ephedra* species, a successful germination in the field may depend on local site (edaphic-climatic) conditions, including soil moisture, salinity, and seasonally cold temperatures [14,15,16]. Under lab-controlled conditions, it appears that the germination of *E. major* seeds can be completed in three weeks without any prior treatment [17]. A more recent greenhouse experiment revealed a high percent of *E. major* seed germination on forest soil, but no effects of cold treatment [18].

Aiming to fill a part of the mentioned knowledge gaps, we tested the seed germination of *Ephedra major* in a garden experiment under different soil and moisture conditions. The main objective of the present pilot study was to explore to what extent the soil quality (in terms of humus content and granulometry) and water availability influence the germination performance of *E. major* seeds, while accounting for their genetic variability.

## 2. Materials and Methods

### 2.1. Study Area

**Figure 1:**
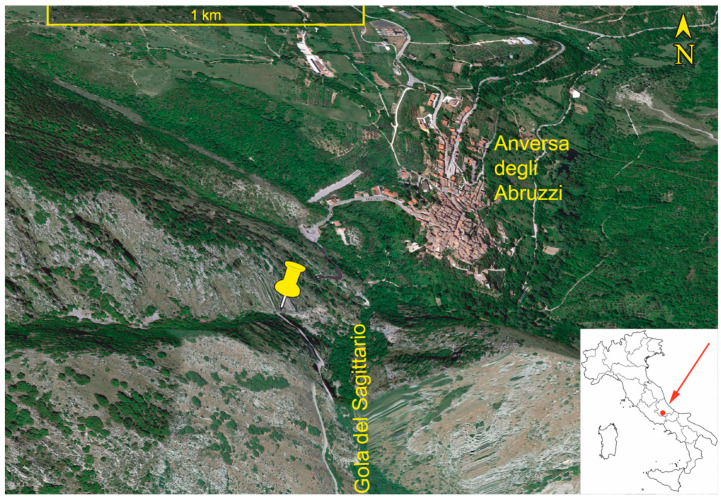
Geographic positioning of Gola del Sagittario within the Italian Peninsula (inset map) and location of the *Ephedra major* seed sampling site (yellow pin). Source: Google Earth Pro imagery shot on the 13 June 2022.

The area under consideration lies in the inner part of the Central Apennines (Adriatic escarpment), within the territory of the Anversa degli Abruzzi municipality (Abruzzo region, central Italy). More precisely, the sampling site is located on a steep, south-eastern slope within the Gola del Sagittario (41°59′26.74″ N and 13°47′54.73″ E; 604 m of elevation) (Figure 1).

By reference to the climatic conditions in the neighbouring areas [19], the study site is characterised by a sub-Mediterranean variant of the temperate bioclimate, with an ombrotype positioned between the lower sub-humid and the upper dry levels. The average annual precipitation ranges between 600 and 800 mm, reaching a peak in late autumn. The continentality, determined by the rainshadow effect of the surrounding mountains, is moderate but ecologically relevant in terms of frosts that hinder the establishment of steno-Mediterranean vegetation.

The geological substrate of the whole study area is made of limestone. The soils in the study sites are attributable to (Lithic) Leptosols or Rendzina, depending on their shallowness i.e., between 0 and 25 cm [20]. These soils are well-drained due to the steep slope and their stoniness. In addition to rock outcrops and gullies that occur scatteredly, small colluvial/debris fans are accumulated on microterraces. The litter cover is thin, discontinuous, and locally absent.

### 2.2. Cone Collection and Drying

**Figure 2:**
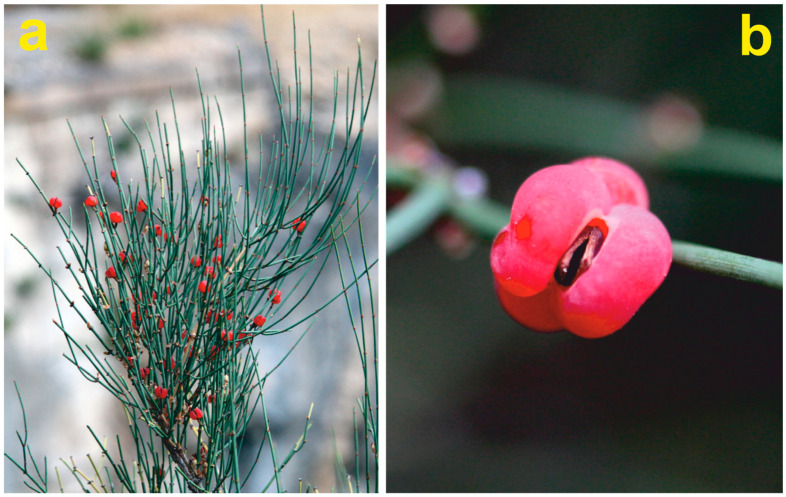
(**a**) Ramet of *Ephedra major* displaying many strobili in the mast year 2008; (**b**) Strobilus close-up showing two inner seeds.

The *E. major* individuals from the study site were observed at least once a year between 2007 and 2019 to monitor their development, dynamics, vegetative spread, and seed set. During the monitoring period, few berry-like cones were produced only occasionally, except for the mast year 2008. In July of that year, many well-developed strobili were observed (Figure 2a).

Fifty partly opened, fleshy strobili displaying well-developed seeds were randomly collected from the study ”population” that actually consisted in many ramets originated from a single (poly-cormus) female individual. From each strobilus, a couple of seeds from the middle cavity were extracted (Figure 2b), ending up with a total of 100 seeds. The selected seeds were stored and naturally dried at ambient temperature in a shady and ventilated place.

### 2.3. Experimental Design

The whole experiment was carried out within the “Carmela Cortini Botanic Garden” of the University of Camerino (Italy), which is situated between the Central Apennine chains at an elevation of 640 m (close to that of the study site). The thermo-pluviometric data recorded at Camerino meteorological station (581 m a.s.l.) during the garden experiment (November 2008 to November 2009) are summarized in Table 1.

In early November 2008, the dried seeds were randomly spread over the soil filling 10 pots (ten seeds per pot) and left undisturbed over the winter, in order to mimic the conditions in the natural habitat. No stimulating treatments (e.g., scarification of the integument, humidification or sinking into hot water) were applied to seeds prior to sowing.

The pots were cylindrical and made of black plastic obtained from recycled polyethylene, with a capacity of 10 litres (24 cm in diameter and 24 cm height). These containers, manufactured by Nuova Pasquini e Bini S.p.a. (Italy), have drainage holes at their bottom and can be used with all types of soil and for both indoor and outdoor cultivation, as they are resistant to UV rays and frost.

The 10 pots were split in two equal, twin groups labelled W0 and W1. All pots were positioned close to each other, partially shaded, and exposed to the same amount of natural rainfall. The only difference between the two groups was that, while the W0 pots received only natural precipitation, the W1 pots were additionally evenly watered in order to keep the topsoil moist and to compensate for the water leak through the holes in the pot bottom.

We collected soil samples in the field from the upper layer (0 to 10 cm), depending on the soil depth. Each of the five pots comprising the group W0 or W1 was filled with a different soil type, namely:

S0–Very shallow (<1 cm), stony soil (Leptosol) of the reference site, drawn from the base of *Ephedra* shrubs;

S1–Similar to S0 but slightly deeper (1 to 2 cm);

S2–Similar to S0 but even deeper (up to 25 cm) and rich in woodland humus, drawn nearby the reference site under tree cover;

S3–Alluvial soil, rich in yellowish, orange, and brown clay (1/3) alternatively superimposed in thin layers, including components of gravel (1/3) and river sand (1/3), collected a little further downstream along the Sagittario River;

S4–Anthropogenic soil, drawn from a former crop area a little further downstream, representing an intimate mixture of clay (1/3), silt (1/3), fine sand (1/6) and slightly coarser sand (1/6), along with traces of small, non-rounded gravel (rock debris and colluvia).

Visits were made at least once a week to check the experimental conditions and to record the germinated seeds. The seedlings of *Ephedra major* emerged in early June 2009 and then had a rapid development, producing between 6 and 10 internodes and, in some cases, even branching. After 10 days of development, the seedlings were unexpectedly and quickly eaten by snails and slugs. The observations continued until November 2009, but no further seed germination occurred.

### 2.4. Data Analyses

Nominal logistic regression was employed to test the effects of soil type and watering on the seed germination rate. The latter was estimated for each treatment as the number of germinated seeds divided by the total number of seeds ascribed to that treatment. Because the interaction term between the two nominal predictors (soil × watering) was not significant (Chi-square = 4.881; *p* = 0.2997), the retained model included only the main effects. The soil type S0 and no watering (W0) were chosen as reference (control) levels of the two treatments. Fisher’s exact test was subsequently applied to pairs of selected levels (S1, S2 and S3) of the significant predictor, without any adjustment to account for multiple testing. A probability threshold of 5% was adopted for deciding whether to reject the null hypotheses or not.

All the statistical analyses were performed in JMP Pro 16.2.0 [23].

## 3. Results

**Figure 3:**
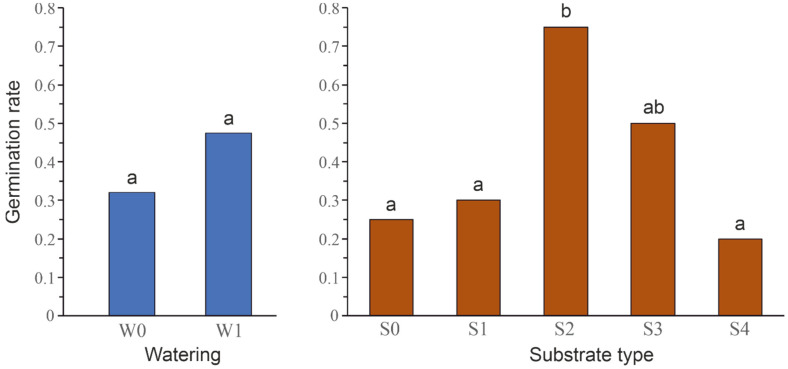
Independent distributions of the germination rate by level of watering treatment (**left**) and soil type (**right**). Different/shared letters correspond to significant/non-significant differences.

The germination rate was higher under the watering treatment, but the difference was barely non-significant (Table 1 and Figure 3).

Overall, the soil type had a significant effect on the germination rate of seeds (Table 2). In particular, only the type S2 had a significant, positive effect on the germination rate, when compared with the types S0, S1 or S4 (Figure 3). In addition, a marginal, positive effect of the type S3, when contrasted to the type S0, was revealed (Table 2).

## 4. Discussions

Soil quality seems to be more important than moisture for the germination success of seeds. The higher germination rate of seeds sown in woodland, humus-rich soils is related partly to the beneficial influence of the absorption of humic substances into seeds [24]. In addition, the humus enhances the microbial activity in the upper soil layer [25], with obvious benefits for germination. Our results are in full accordance with the high germination rates of *E. major* seeds observed on forest soil by Mofid Bojnoordi et al. [18]. All these clearly suggest a positive relationship between the amount of soil organic matter and the success rate of *E. major* seed germination. If the observed experimental patterns hold true in the field, the *E. major* seeds are expected to display a higher germination success rate under the open canopy of woodlands compared to open, rupicolous habitats, since seeds can benefit from higher humus availability and reduced evapotranspiration (risk of desiccation).

The positive, although marginal, effects of watering and soils featuring clay layers suggest that higher rates of seed germination could possibly be reached under optimal moisture conditions. This hypothesis is supported by a previous greenhouse experiment revealing larger germination rates in soaked/washed seeds compared with that observed in untreated seeds [18]. However, this presumed reproduction gain can be completely counteracted by herbivores (e.g., slugs) feeding on *Ephedra* seedlings or root rot fungi that benefit from increased soil moisture [26]. That was very likely determined by the high amount of rainfall registered in June 2009. On the other hand, no effect of seed soaking in hot water on the germination rate of *Ephedra nebrodensis* (synonym of *E. major*) was reported [17], but that experiment was carried out in artificial conditions i.e., seeds placed on filter papers and kept into a heated germinator.

Since the inexorable differences in seed viability were controlled by their random assignment to treatments, the experimental low germination success on humus-poor, very shallow soils raises concern about the persistence of *E. major* populations in open, rupicolous habitats, where they play an important protective role against soil erosion and displacement of loose substrates. Moreover, the germination performance we observed “ex situ” may be superior to that reachable in natural habitats under similar soil and moisture conditions, as noticed, for instance, in a relictual population of *Ephedra distachya* in Slovakia [27]. All these concerns are amplified by the rare occurrence of *E. major* in distant, residual, small-sized populations that sometimes (like the present case) are reduced to a single genetic individual [28]. Despite the fact that *E. major* was not included in the most recent Red List of vascular species in Italy [29] and is currently not protected by law, it is affected by several anthropogenic threats [30] that can drastically reduce the chance of sexual reproduction and may lead to local extinction [28]. In fact, relying exclusively on vegetative propagation from root suckers for the maintenance of *Ephedra* populations comes with a series of negative consequences, such as the decline in genetic variation [31].

## 5. Preliminary Conclusions and limitations

The outcomes of this pilot experiment suggest that the success of *E. major* seed germination increases with the amount of humus (and possibly water) in the underlying soil. However, this relationship is almost certainly not monotonic and should be subsequently validated through field experiments in different vegetation and habitat types. To our knowledge, this is the first study dealing exclusively with the ecology of seed germination in *Ephedra major* under open, close-to-nature conditions.

The power of the employed statistical analyses was limited by the small number of replications in each of the two experimental treatments. This was probably the reason for observing only marginal effects of the clay-layered soil and watering on the germination success of *Ephedra major* seeds.

## Figures and Tables

**Table 1:** Monthly temperature and precipitation data recorded at Camerino meteorological station (581 m a.s.l.) from November 2008 to November 2009 (source: [21,22]).

Month	Precipitations (mm)	Rainy Days (Counts)	Mean of Minimum Temperatures (°C)	Mean of Maximum Temperatures (°C)
November 2008	121.4	12	6.0	12.1
December 2008	205.4	14	2.2	7.5
January 2009	59.4	9	1.4	5.8
February 2009	61.2	12	0.8	6.7
March 2009	77.0	13	4.7	11.8
April 2009	80.0	12	8.5	15.6
May 2009	17.8	2	13.8	23.2
June 2009	158.4	9	15.5	23.3
July 2009	83.0	2	18.3	28.3
August 2009	68.4	5	18.5	29.2
October 2009	65.4	7	14.8	23.2
November 2009	97.6	10	9.1	16.9

**Table 2:** Effect term coefficients and goodness-of-fit statistics associated with the model estimating the log odds of seed germination (Yes/No) by level of watering and soil type.

Term	Coefficient	Chi-Square	Prob.	Whole Model Statistics
Intercept	−0.464	4.03	0.0448	Chi-sq. = 20.69 *p* = 0.0009 R-sq.(U) = 0.154
Soil type [S1]	−0.418	0.87	0.3519	
Soil type [S2]	1.605	11.57	0.0007	
Soil type [S3]	0.973	3.84	0.0501	
Soil type [S4]	−0.464	1.20	0.2725	
Watering [W1]	0.411	3.15	0.0762	

## Data Availability

The datasets generated for this study are available on request to the corresponding author (KC).
